# Enhanced Specificity of Multiplex Polymerase Chain Reaction via CdTe Quantum Dots

**DOI:** 10.1007/s11671-010-9797-5

**Published:** 2010-09-30

**Authors:** Gaofeng Liang, Chao Ma, Yanliang Zhu, Shuchun Li, Youhua Shao, Yong Wang, Zhongdang Xiao

**Affiliations:** 1 State Key Laboratory of Bioelectronics, School of Biological Science and Medical Engineering, Southeast University, SiPaiLou2#, 210096, Nanjing, China

**Keywords:** PCR, Quantum dots, Specificity

## Abstract

Nanoparticles were recently reported to be able to improve both efficiency and specificity in polymerase chain reaction (PCR). Here, CdTe QDs were introduced into multi-PCR systems. It was found that an appropriate concentration of CdTe QDs could enhance the performance of multi-PCR by reducing the formation of nonspecific products in the complex system, but an excessive amount of CdTe QDs could suppress the PCR. The effects of QDs on PCR can be reversed by increasing the polymerase concentration or by adding bovine serum albumin (BSA). The mechanisms underlying these effects were also discussed. The results indicated that CdTe QDs could be used to optimize the amplification products of the PCR, especially in the multi-PCR system with different primers annealing temperatures, which is of great significance for molecular diagnosis.

## Introduction

The PCR has become one of the most popular tool for molecular biology and pathogen detection as a high sensitive technique [1-3], but it still requires careful optimization on reaction conditions in order to eliminate nonspecific products in most PCR experiments, especially in multiplex PCR (multi-PCR). Optimized parameters could include the type and concentration of the enzyme, reaction buffer content, time and temperature of the annealing/extension process, primer design and use of a rapid heating–cooling response PCR machine [4,5]. It was reported that a variety of organic chemicals, including single-stranded DNA-binding proteins (SSBs) [6], amides and betaine [7], imidazole, tetramethylammonium chloride (TMAC) and TMAC derivatives [6-9], could act as accelerants in PCR mixture to increase yield and specificity.

As a novel material, nanoparticles (NPs) have many important applications in biology, for example in drug delivery, biomolecules separation, cancer thermal therapy, ultrasensitive biosensor and live cell imaging [10-15]. Recently, gold nanoparticles have been reported to be able to reduce the level of nonspecific products even at lower annealing temperature [9,16,17]. Li et al. [16] found that Au NPs can improve the efficiency of PCR and attributed the enhancement to the excellent heat conductivity of Au NPs. But some researchers reported some discriminating conclusions [17-19]. Yang et al. [18] evaluated the function of Au NPs in PCR systems and suggested that a complex interaction between Au NPs and native Taq DNA polymerase was accounted for the effects of Au NPs on PCR. Vuet et al. [19] demonstrated that AuNPs tended to favor smaller fragments of PCR products than larger ones owing to the different polymerase adsorptions. Possible reasons concerning these previous discrepant results might be referred to different nanoparticles used in terms of surface modification and some other different experimental conditions.

Quantum dots, as a new kind of fluorescent material, have many excellent features, such as size-tunable emission, broad excitation profiles and narrow/symmetric emission spectra, high photostability. The new generations of QDs have far-reaching potential for the study of intracellular processes at the single-molecule level, high-resolution cellular imaging, long-term in vivo observation of cell trafficking, tumor targeting and diagnostics [20]. CdTe QDs have the advantage of fluorescence at different wavelengths and could be used as potential fluorescent materials in real-time PCR. Here, CdTe quantum dots (QDs) were employed as a novel assisting agent to systematically investigate the effects of nanoparticles on PCR. First, two-round PCR was used to amplify a 310-bp fragment from *Triticum aestivum* genomic DNA and then the performance of PCR via the QDs was evaluated by multi-PCR and real-time PCR. The results showed that CdTe QDs could reduce the formation of nonspecific products at an appropriate concentration, but inhibit the amplification when using an excessive amount of QDs in the multi-PCR mixture. The mechanisms underlying these effects were also discussed.

## Materials and methods

### Preparation of CdTe QDs

**Figure 1:**
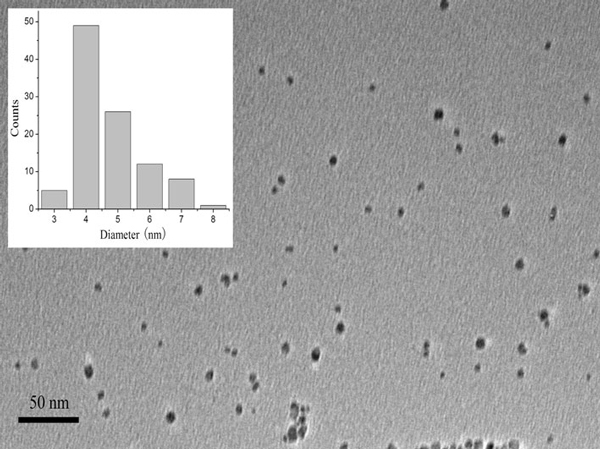
**TEM image of the CdTe QDs used in the experiments**.

Aqueous solutions of monodisperse CdTe NPs were synthesized as described previously [21]. Briefly, 2.4 mmol of CdCl_2_·2.5H_2_O was dissolved in 125 ml of water, and 5.76 mmol of TGA was added under stirring, and the pH of above mixture was adjusted to 11.4 using NaOH. After the solution was deaerated by Ar bubbling for 30 min, H_2_Te was generated by dropping 10 ml of 0.5 M H_2_SO_4_ into freshly prepared NaHTe solution. The as-formed orange solution was then refluxed to control the growth of the CdTe nanoparticles to the desired size. The as-synthesized CdTe nanoparticles were precipitated by 2-propanol followed by centrifugation at 8,000 rpm for 5 min at room temperature; then, the precipitate was redispersed in water. The above process of precipitation and centrifugation was repeated twice to purify the CdTe QDs. The average size of CdTe QDs used in the experiments was 4.5 nm (Figure 1).

### The Template DNA and Primers

**Figure 2:**
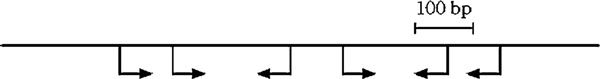
**Map including the localizations of PCR primers on *Triticum aestivum* 18S rDNA gene**.

Genomic DNA was isolated from fresh leaves of *Triticum aestivum* using Wizard^®^ genomic DNA kit (Promega, USA), according to the manufacturer's protocol. The target gene is 18S rDNA gene for *Triticum* species. Figure 2 is an illustration of multiplex PCR with three forward primer and three reverse primers. Primers were designed by primer premier 5.0 and synthesized by Sangon (Shanghai, China), including the forward primer 5'-ACGATCAGATACCGTCCTA-3' and the reverse primer 5'-ACAAATCGCTCCACCAAC-3' for amplification of the 310-bp templates. Other primers for the amplification of templates of various lengths were synthesized by the same company, and the sequences are given in Table 1.

**Table 1:** Primers for 18S rDNA templates

**Name**^**a**^	Sequence (5' to 3')
390F	AGCCTGAGAAACGGCTAC
434F	CAAATTACCCAATCCTGAC
995F	ACGATCAGATACCGTCCTA
542R	GATCCTCGTTAAGGGATTTA
1036R	TCGGCATCGTTTATGGTT
1304R	ACAAATCGCTCCACCAAC

^a^In primer names, the number indicates the position of primer on the template. The letter (F or R) indicates the forward or reverse primer, respectively

### The Conventional PCR and Real-Time PCR Systems

The ABI2720 PCR machine and the ABI 7500 real-time PCR System (Application Biosystems, Foster, USA) were used in this study. For specificity test, two-round PCR was used in which the PCR products from the first round were used as the DNA template for the second-round PCR [9,22]. The specificity of amplification was also tested using multiplex PCR in which two reverse primers and two different forward primers were used. Each conventional PCR was carried out with 50 pg–15 ng of DNA template, 0.25 μM of each primer and 12.5 μl of BioTaq master mix (BioTeke, Beijing, China) in a final volume of 25 μl. As a parallel assay, rTaq DNA polymerase (TaKaRa, Dalian, China) was used in the study. PCR was run in an ABI 2720 cycler (applied Biosystems) with 35 cycles of 15 s denaturation at 95°C, 35 s annealing at 50°C, followed by 50 s extension at 72°C. Cycling was started after an initial denaturation at 95°C for 5 min and ended with a final extension at 72°C for 5 min. Only the annealing temperature was changed for the experiments at different annealing temperatures. The PCR products were collected and submitted to electrophoresis in 1.6%/TBE agarose gel.

ABI 7500 real-time PCR system and its SDS software (version 1.40) were used to evaluate amplification efficiency. The 20 μl reactions were carried out including 250 pg of DNA template, 0.2 μM of each primer and 1× SYBR^®^ Green PCR master mix (TaKaRa, Dalian, China). The PCR temperature is as follows: 5 min at 95°C; 40 cycles of 15 s at 95°C, 1 min at 60°C.

## Results and Discussion

### Effects of CdTe QDs Concentrations on the Specificity of PCR

**Figure 3:**
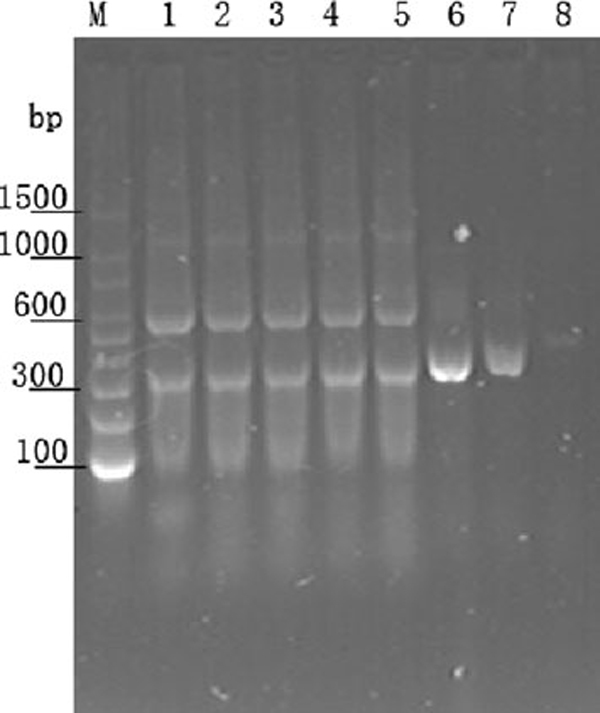
**The effect of different concentrations of CdTe QDs on the specificity of PCR, amplification is a 310-bp 18S rDNA fragment from *Triticum aestivum* genomic DNA**. *Lane M* DNA marker, *lane 1* 0 nM, *lane 2* 0.1× PBS, *lane 3* 10 nM, *lane 4* 20 nM, *lane 5* 40 nM, *lane 6* 60 nM, *lane 7* 80 nM, *lane 8* 100 nM.

Specificity of PCR amplification is affected more frequently by numerous factors, such as the size, complexity and copies of target DNA or RNA and primer design. In this study, we first amplified a 310-bp fragment from *Triticum aestivum* genomic DNA, and the PCR products from the first round were used as template for the second-round PCR, which was employed as a model system for the analysis of effect of QDs on PCR amplification. Figure 3 showed the effect of different concentrations of CdTe QDs on the specificity of PCR at an annealing temperature of 55°C. Compared with lane M (marker), lane 1–5 (control without QDs or inadequate QDs) showed a broad distribution of nonspecific amplification bands. Lanes 3–8 showed the PCR products in the presence of 10, 20, 40, 60, 80 and 100 nM CdTe nanoparticles, respectively. In the presence of 60 nM CdTe QDs in lane 6, only a single specific band with a size corresponding to the 310 bp target sequence was observed and nonspecific bands disappeared. Similar enhancement was not observed in control experiment by the addition of 0.1× PBS to the reaction mixture (lane 2). It is obvious that an increase in the QDs enhanced the specificity of PCR products. However, with the concentration of QDs increasing, the brightness of 310 bp specific band (lane 7) becomes weaker than the products at the concentration of 60 nM. Lane 8 suggested that PCR was inhibited obviously when excessive QDs were added into PCR mixture. Therefore, QDs concentration is a critical factor for improving PCR amplification.

**Figure 4:**
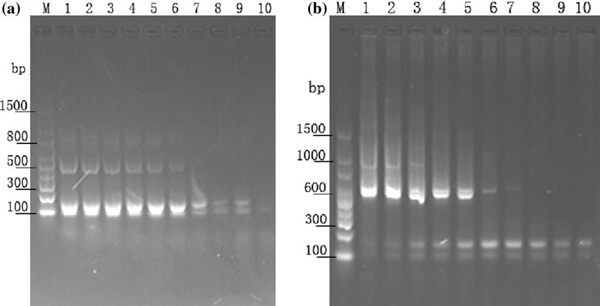
**Effect of CdTe QD on different amplicon lengths**. **a** Multiplex PCR assay was carried out using a Triticum aestivum genomic DNA template with two forward primers (390F, 434F) and one reverse primer (542R). *Lane M* is marker; *lane 1* is control without CdTe QDs. *Lane 2* contains 10 nM CdTe nanoparticles, *lane 3* 20 nM, *lane 4* 30 nM, *lane 5* 40 nM, *lane 6* 50 nM, *lane 7* 60 nM, *lane 8* 70 nM, *lane 9* 80 nM, *lane 10* 100 nM. **b** Multi-PCR similar to Figure 4a but with two forward primers (390F, 434F) and two reverse primers (542R, 1036R). The concentration of CdTe QDs is identical to Figure 4a.

Enhancement of specificity was validated by multi-PCR experiments in the presence of semiconductor CdTe QDs, which further revealed that the use of QDs with the concentrations ranging from 60 to 80 nM in the PCR reagent can effectively eliminate the nonspecific amplified products (Figure 4). The results also showed that more than 100 nM QDs led to PCR inhibition. This effect for improvement in PCR specificity was also observed using different primer–template pairs employed to amplify four fragments in multi-PCR system at an annealing temperature of 50°C (Figure 4b). Despite widespread use, multiplex PCR is still baffled by inherent problems such as preferential target amplification and nonspecific amplifications. When appropriate QDs were added into PCR mixture, the performance of PCR was improved significantly. As shown by semi-multiplex PCR with two forward primers and one reverse primer (Figure 4a), only two specific bands were observed, and all nonspecific bands disappeared when an appropriate concentration of CdTe QDs (60 nM) was added to the PCR mix. An excessive amount of QD may inhibit the specific products, as shown by the attenuation of the specific band in lane 10. Similar phenomena were observed in another multi-PCR system with two forward primers and two reverse primers (Figure 4b). This study suggested the feasibility of improving specificity by using of QDs in multiple PCR systems. It should be noted that an appropriate concentration of QDs can improve the specificity of PCR, while an excessive concentration could inhibit the amplification process.

In previous experiment, another interesting phenomenon was observed (Figure 4). The specific bands 647 and 603 bp gradually disappeared with the increase in QDs concentration while that of the short target fragments of 153 and 109 bp become gradually brighter, and all bands disappeared in 100 nM lane. This indicates that the CdTe QDs could optimize the PCR process through modulating the concentration of the component in PCR mix.

### Reverse Effects of CdTe QDs by BSA and Polymerase

**Figure 5:**
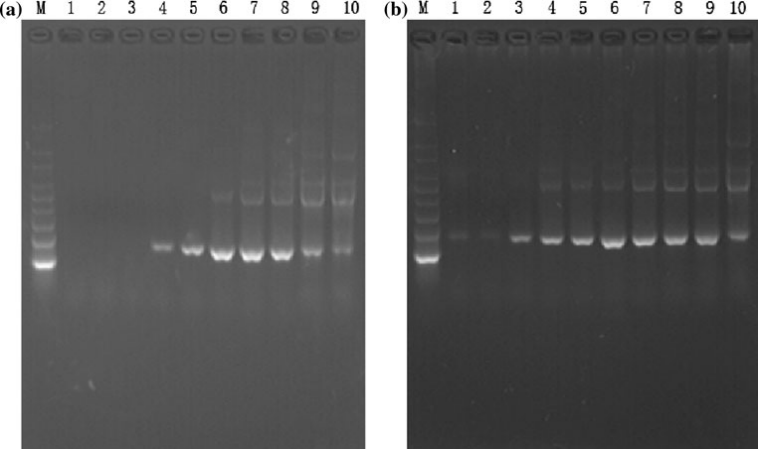
**The effects of CdTe QDs on PCR were reversed by adding BSA or extra polymerase**. **a** The concentration of CdTe QDs in *lanes 1*–*10* was 100 nM. Increasing BSA concentration gradually reversed the effect of QDs. **a** BSA concentrations: *lanes 1*–*10* 10, 20, 30, 70, 150, 300, 600 μg/ml, 1, 2, 5 mg/ml, respectively. **b** extra polymerase reversed the effect of CdTe QDs. **b** Taq polymerase concentration: *lane 1–10* 0.3 U, 0.5 U, 1.0 U, 1.5 U, 2 U, 3 U, 4 U, 5 U, 6 U, and 8 U, respectively.

It was found that increasing the concentration of primers or template did not alter the effects of QDs (results not shown), but adding bovine serum albumin (BSA) or increasing the concentration of Taq polymerase could completely reverse the effects of CdTe QDs on PCR. As shown in Figure 5a, with 100 nM CdTe QDs as a control PCR that inhibits the reaction entirely, by adding different concentrations of BSA, nonspecific amplification bands reoccurred despite a high annealing temperature. The results indicated that BSA can completely reverse the effect of CdTe QDs. Furthermore, the similar phenomenon can be observed by increasing the concentration of polymerase in the reaction, as shown in Figure 5b.

### Effect of QDs on PCR Efficiency

**Figure 6:**
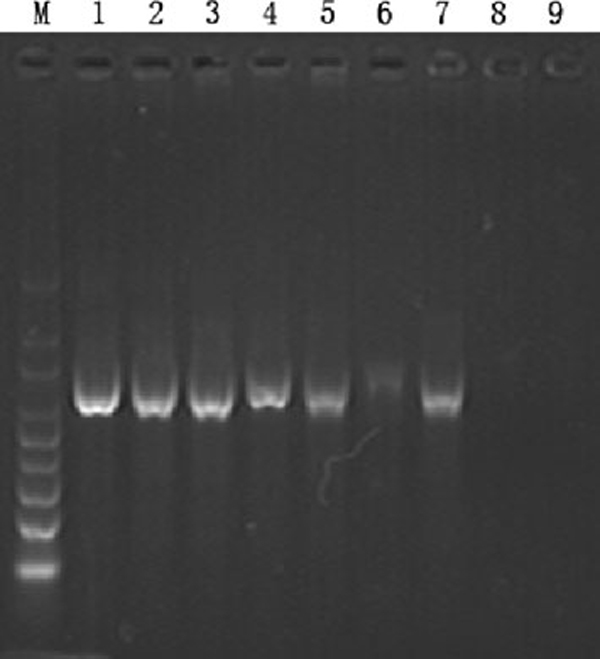
**Efficiency experiment of the QDs in PCR**. *Lane 1*–*3*, 1/2 time of standard PCR; *lane 4*–*6*, 1/4 time of standard PCR; *lane 7*–*9*, 1/10 time of standard PCR. *Lanes 1*, *4* and *7* contained CdTe QDs 20 nM; *lane 2*, *5* and *8* contained CdTe QDs 40 nM; *lane 3*, *6* and *9* contained CdTe QDs 60 nM, respectively.

**Figure 7:**
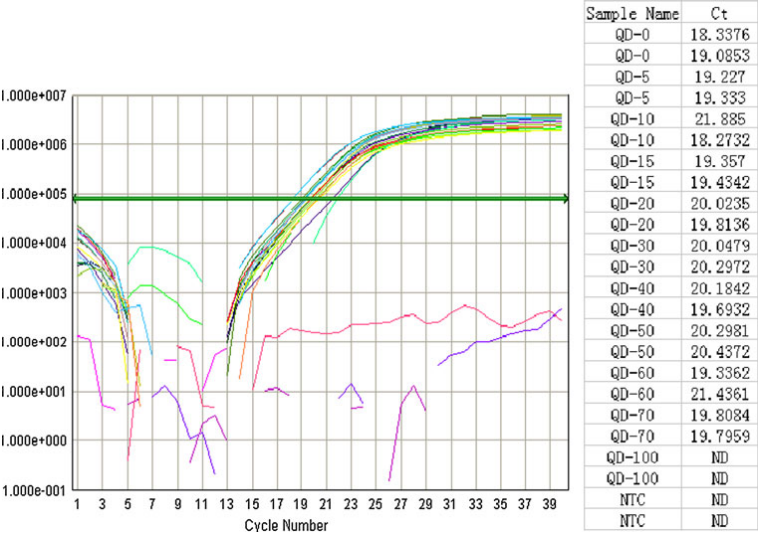
**The effects of QDs on PCR efficiency was validated in real-time PCR system**. The number indicates the corresponding concentration in nM level of QDs adding to the PCR mix. The results suggested that low concentration of CdTe QDs had no obvious effect on the PCR process but excess QDs result in inhibition.

The electrophoresis and real-time PCR were used to study the effects of CdTe QDs on efficiency of PCR. Figure 6 shows that the yield of the PCR product was reduced when the length of time in all steps of the standard PCR protocol was reduced. CdTe QDs at the optimized concentration were included in the time-shortened PCR system, and there was a clear correlation between the increased yield of the PCR product and the amplification time. Lane 1–3, lanes 4–6 and lane 7–9 show the results with different concentrations of CdTe QD added to PCR mixture using 1/2, 1/4 and 1/10 of the amplification time of the standard PCR, respectively. The QDs did not display any obvious improvement in the efficiency of the PCR. Conversely, the existing strong bands in lanes 1–3 became weakened in lane 4–6 and disappeared in lanes 8, 9 with the time-shortened PCR system. The results were further validated by the real-time PCR. As shown in Figure 7, there was no obvious improvement in the efficiency of the PCR after adding different concentrations of CdTe QDs, even inhibit the PCR process at 100 nM, which indicates that CdTe QDs could not improve the efficiency of PCR. This result was different from that reported by Li [16,18] but in accordance with the works by Wang [17], Vu [19] and Wan [23].

Why the QDs could improve the specificity of the PCR? There are two possible reasons for the question. On the one hand, the improvement effect of the QDs to the specificity of the PCR could be attributed to the similar optimization mechanism of the single-stranded binding protein (SSB), which selectively binds to the single-stranded DNA rather than double-stranded DNA and then minimizes the mispairing between the primers and the templates in the PCR system [6,24]. On the other hand, the interaction between the DNA polymerase and the QDs might enhance the specificity of PCR. The possibility was confirmed by our experiments. The effects of CdTe QDs on PCR did not alter by increasing the concentration of template or primers, but were completely reversed by increasing the concentration of Taq polymerase in the PCR mixture. This suggested there was an interaction between QDs and DNA polymerase. Additionally, the effects of CdTe QDs on PCR can be reversed by adding BSA as a competitor with QDs in the reaction, strongly implying that the action of CdTe QDs in PCR might be through the adsorption and desorption of polymerase. The adsorption of polymerase by CdTe QDs would lead to a reduction in effective polymerase concentration. When polymerase concentration become rare limited, only the target PCR products, which were annealed with primers most efficiently, would be amplified preferentially. While PCR with excessive polymerase concentration tends to produce high molecular weight nonspecific products, and at lower polymerase concentrations might be anticipated to show lower processivity and bias toward smaller fragments [25]. This was the possible reasons why the PCR products were shifted from the larger amplicon to the smaller amplicon observed in Figure 4. Finally, the specific products were also eliminated when adequate QDs are added into the reaction mixture. This phenomenon was validated with rTaq and power Taq polymerase in our study.

As a polypeptide molecule, DNA polymerase can be interacted with other biomolecules, such as protein, nucleic acid. The present results indicated that CdTe QDs could regulate the effective polymerase concentration in PCR process. Being different from other binding factors of protein, the binding process of CdTe QDs can be regulated and reversed by adding a competitor, i.e., BSA. The present experiments showed that low concentration of CdTe QDs had no obvious effect on the PCR process but excess CdTe QDs would result in the repression of PCR process, and only appropriate concentration of QDs could improve PCR specificity. Inspired by these results, we propose a possible mechanism that CdTe QDs optimize the PCR process by regulating the dynamic equilibrium between DNA polymerase and QDs. A central idea of the hypothesis was from the reversible binding of CdTe QDs and free polymerase. As well known, the polymerization is consisted of two processes in PCR system, i.e., the binding of polymerase with primer–template junction (PTJ) and extension of the binding. Higher concentration polymerase might have more opportunity to bind PTJ, which squint toward increase in the average length of products synthesized on a long template, result in more nonspecific products [25]. By regulating the dynamic equilibrium of binding of polymerase with primer–temples junction, the PCR process and DNA synthesis could be modulated. Whereas low concentration CdTe QDs have no effect on the equilibrium and higher concentration CdTe QDs might inhibit the interaction of primer–template–polymerase. Considering that PCR is a very complex process, intensive studies should be carried out in order to reveal the underlying mechanism of QD-PCR.

## Conclusion

As gold and other nanoparticles, the synthesized carboxyl-based CdTe QDs did indeed effectively improve the PCR specificity. In the normal PCR and multi-PCR system, the specific PCR products were achieved when the different concentrations of QDs were added into the PCR system. The optimal concentration of the added QDs was obtained in present multi-PCR systems. Based on the research results, the QDs could be used as a useful additive in the PCR, especially multiplex PCR. The ability of QDs to improve the specificity of PCR is advantageous for the expansion of its biological applications. Being the complexity of PCR process, intensive studies should be carried out in order to reveal the in-depth mechanism of QDs in multiplex PCR.
